# Primary mediastinal synovial sarcoma: a case report and review of the literature

**DOI:** 10.4076/1757-1626-2-6948

**Published:** 2009-08-07

**Authors:** Benjamin Henninger, Martin Freund, Bettina Zelger, Daniel Putzer, Hugo Bonatti, Ludwig Müller, Michael Fiegl, Christian Geltner

**Affiliations:** 1 Department of Radiology, Innsbruck Medical UniversityAnichstraße 35, 6020 InnsbruckAustria; 2 Institute of Pathology, Innsbruck Medical UniversityAnichstraße 35, 6020 InnsbruckAustria; 3 Department of Nuclear Medicine, Innsbruck Medical UniversityAnichstraße 35, 6020 InnsbruckAustria; 4 Department of Surgery, University of Virginia Health ServicesCharlottesville, VAUSA; 5 Department of Cardiac Surgery, Innsbruck Medical UniversityAnichstraße 35, 6020 InnsbruckAustria; 6 Department of Oncology, Natters HospitalIn der Stille 20, 6161 NattersAustria; 7 Department of Pulmonology, Natters HospitalIn der Stille 20, 6161 NattersAustria

## Abstract

Primary mediastinal synovial sarcoma is a rare malignancy with only a few cases reported so far. A 56-year-old woman was admitted to our hospital for an investigation of a nodule in the left middle lung on chest radiography. Computed tomography revealed a mediastinal mass first described as a solitary fibrous tumor. The diagnosis of synovial sarcoma was established by computed tomography-guided percutaneous needle biopsy. Work up showed no metastasis to distant organs or contralateral pleural cavity. The mass was surgically resected; pathological and immunohistochemical analyses confirmed the diagnosis of a monophasic spindle cell synovial sarcoma probably originating from phrenic nerve. The patient received adjuvant chemotherapy and radiation and is free of recurrence after a follow up of 16 months.

## Introduction

Soft tissue sarcomas (STS) are a heterogeneous group of neoplasms. They account for less than 1% of all adult malignancies [1]. Synovial sarcoma is a malignant mesenchymal neoplasm that has been reported in children and adults equally involving men and women. It accounts for up to 10% of all histological types of soft tissue sarcomas [2], is unrelated to synovium and can occur in almost any part of the body. Survival rates of this malignancy have been reported to range between few months and many years. Therapy includes surgical resection, radiation and chemotherapy with various agents having been used. Mediastinal synovial sarcoma is very rare, only a few cases have been reported in the recent years. We report a new case of this entity.

## Case presentation

**Figure 1.**
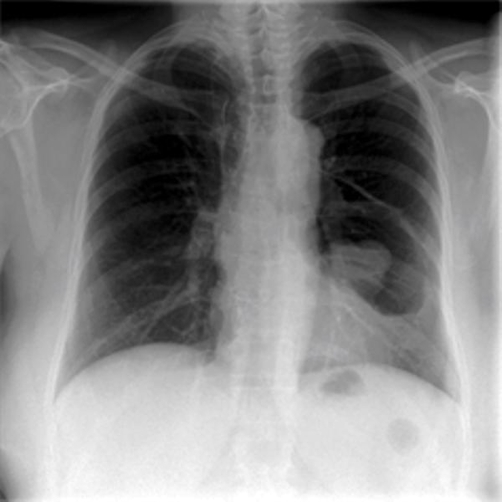
Chest radiograph demonstrating a mass in the left middle field.

**Figures 2 and 3.**
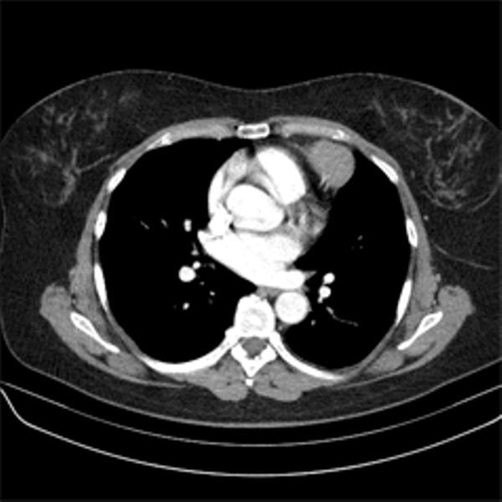

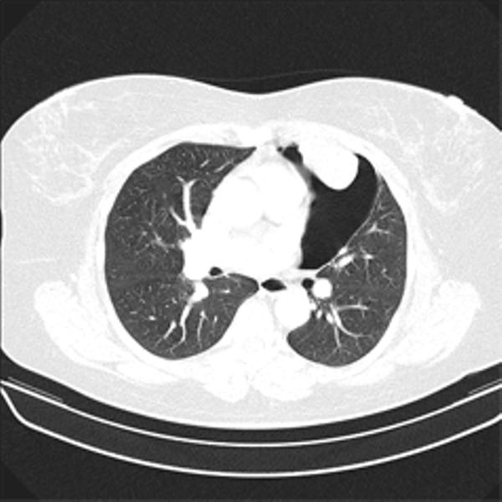
Chest computed tomography showed an 3 × 5 cm measuring intrathoracic tumor with broadly based contact to the pericardium, surrounded by pneumatocele. It was identified as an extrapulmonary, mediastinal benign solitary fibrous tumor.

A 56-year-old Caucasian woman was referred to our hospital after a chest X-ray showed a dense mass in the left middle lung (Figure 1). The patient had been complaining of increasing dyspnea during exercise since one year and symptoms of chronic bronchitis for several weeks, which was resistant to therapy. No family history of cancer was reported and the patient had no history of smoking. Clinical examination was without any pathological findings except for dullness and reduced breath sounds over the right lung. Chest computed tomography (CT) showed a 3.0 × 5.0 cm intrathoracic tumor, which was surrounded by pneumatocele (Figures 2 and 3). This seemed most consistent by morphological criteria with an extrapulmonary, mediastinal benign solitary fibrous tumor. No metastases where found in lymph nodes or distant organs.

Bronchoscopic examination revealed a constriction of the left upper lobe but no endobronchial tumor. CT-guided percutaneous needle biopsy was performed and histological H&E examination at the Institute of Pathology Medical University Innsbruck showed a monomorphic cell rich spindle cell proliferation with mild nuclear atypia. The characteristic H&E morphology of the core biopsy together with homogenous vimentin positivity, bcl-2 positivity and focal keratin (CAM 5.2, CK7, CK19) as well as EMA positivity together with the absence of S-100 Protein (rules out malignant peripheral nerve sheath tumor with focal keratin expression), calretinin (rules out mesothelioma together with the clinical picture and the negative history for asbest exposure) and CD34 (rules out solitary fibrous tumor) led to the diagnosis of a monophasic synovial sarcoma.

**Figure 4.**
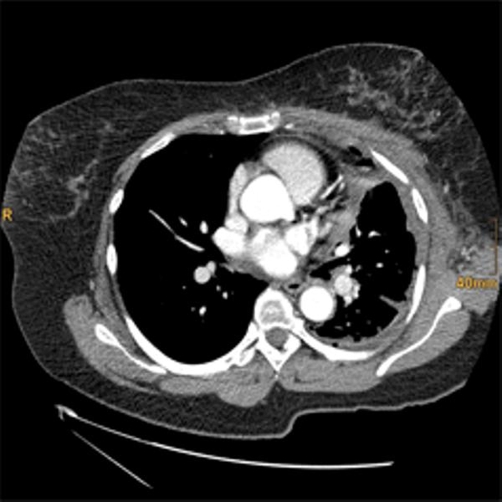
Chest computed tomography after resection of the tumor through a thoracotomy. This image shows volume reduction and fluid accumulation with no signs of relapse.

**Figure 5.**
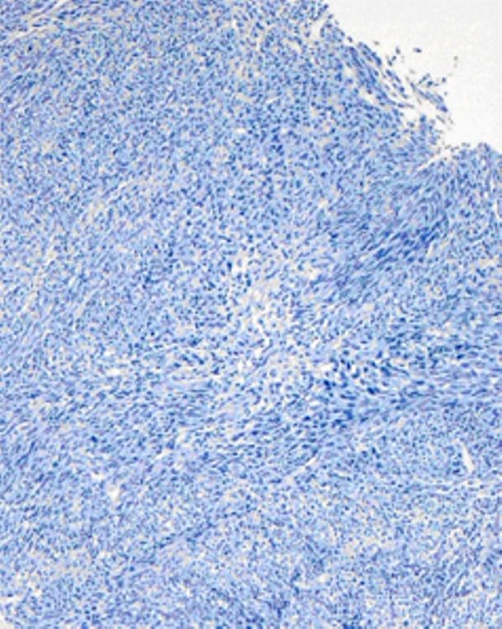
Dense cellular spindle cell proliferation with fascicular growth pattern and nuclear atypia. No glandular biphasic pattern. H&E, 100×.

**Figure 6.**
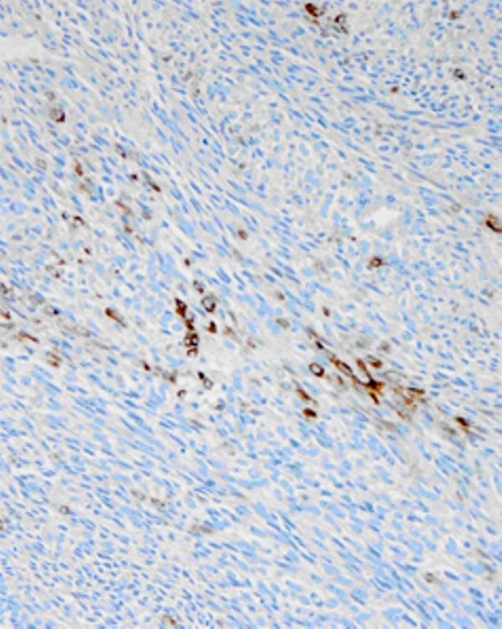
Focally strong keratin positivity. Immunohistochemistry with Cam 5.2, 200×.

Discussion at the multi-disciplinary bone and soft tissue tumor board (MUI) as well as in the lung and mediastinal tumor board (MUI) decided to do primary surgery as there was no spread of disease, no metastases on X-ray and CT. The tumor was surgically resected by thoracotomy (Figure 4). The mediastinal mass was in close association with the phrenic nerve, which had to be resected together with a fragment of the diaphragm. The defect was closed with interrupted sutures. On gross examination it presented as a polycyclic well circumscribed with 7 cm in greatest diameter. The cut surface was whitish-grey and of soft consistency. Histological and immunohistochemical examination confirmed the diagnosis of synovial sarcoma (Figures 5 and 6). The final tumour was staged as pT2b N0 M0 R0. The patient recovered without any complications from surgery and received four cycles of adjuvant chemotherapy (100 mg Doxorubicin and 3000 mg Ifosfamid) and thoracic radiotherapy two months later. The patient is currently free of recurrence after a follow-up of 16 months.

## Discussion

Synovial sarcoma originates in the deep soft tissue and commonly presents as an asymptomatic slow growing mass. In almost 60% of cases the neoplasm is located in an extremity; less than 20% occur in the trunk [2]. Synovial sarcoma has been described in numerous nonsynovial locations including the abdomen, the heart, the prostate, the colon, the orbit, the pleura and the lung. Despite its name synovial sarcoma does not represent synovial origin. According to the “WHO 2002 Classification of Tumours: Tumours of Soft Tissue and Bone” it is classified as of uncertain histogenesis [3].

In a study by Spillane et al. the overall 5-year survival rate for STS was 57%. They also demonstrated that an age greater than 20 years at diagnosis and the trend in size (≥ 5 cm) were associated with a significantly worse prognosis. Their survey assessed one hundred and fifty patients [4]. In our case both prognostic factors (> 20 years of age, size of tumor ≥ 5 cm) suggests a relatively poor prognosis for the patient. Deshmukh et al. proposed that synovial sarcoma patients presenting with a primary tumor larger than 5 cm should be considered for more aggressive surgery in combination with radiotherapy or chemotherapy [5].

Only a few other cases of synovial sarcoma of the mediastinum have been reported [6-13]. All cases are summarized in Table 1. Of note, the number of diagnosed thoracic synovial sarcoma has recently increased. The differential diagnosis includes various neoplasms of the chest such as localized fibrous tumors of the pleura, malignant mesothelioma, primary and metastatic lung neoplasms, thymoma and other rare primary parenchymal sarcomas.

**Table 1.** 

Authors	Title	Journal	Cases	Age	Sex	Clinical presentation	Radiological presentation	Therapy	Outcome	Comments
Jeganathan et al. [6]	Primary mediastinal synovial sarcoma.	Ulster Med J 2007; 76:109-111	1	59	m	non-specific abdominal pain	large mass in left thoracic cavity, adjacent to the mediastinum	surgery	disease free 18 months post-operative	F-18 FDG PET scans were performed with increased uptake of the tumor
Gotoh et al. [7]	Synovial sarcoma of the mediastinum: report of a case.	Surg Today 2004; 34:521-524	1	50	m	anterior chest pain	10 × 8 cm mass in the right anterior mediastinal space, no sign of chest wall invasion or disseminated lesions	surgery, chemotherapy (ifosfamide)	patient is alive 9 month after operation	local recurrence in the right pleural cavitiy and metastasis to mediastinal lymph node were detected 9 months postoperatively
Witkin et al. [8]	A biphasic tumor of the mediastinum with features of synovial sarcoma. A report of four cases.	Am J Surg Pathol 1989; 13:490-499	4	range 40-73	m	localized symptoms: hoarseness and cough / dyspnea and palpitations / hemoptysis	solitary mediastinal masses	surgery and radiotherpay / surgery, chemotherapy and palliative radiotherapy / 2 had only surgery	3 patients died of their disease 14 months / 4 years / 10 months after diagnosis	-
Trupiano et al. [9]	Mediastinal synovial sarcoma: report of two cases with molecular genetic analysis.	Ann Thorac Surg 2002; 73:628-630	2	67 / 30	m / f	first patient had chest pain and shortness of breath / second presented incidental	9.0 cm soft tissue mass extending over the cardiac apex / 17.0 cm anterior mediastinal mass	surgery (partial pericardectomy), radiation / partial resection (pericardectomy) and wedge resection of the left upper lobe of the lung, multiagent chemotherapy	alive after 18 months after diagnosis / expired 10 months after inital presentation	-
Hsieh et al. [10]	Synovial sarcoma of the mediastinum.	Zhonghua Yi Xue Za Zhi (Taipei) 2002; 65:83-85	1	11	m	facial edema, flushing, poor appetite and fatigue	widening superior mediastinum with increased densitiy, CT showed a big mass in the right superior mediastinum with chest wall invasion	surgery, chemotherapy, radiation	alive 2 years after diagnosis	-
Suster et al. [11]	Primary synovial sarcomas of the mediastinum: a clinicopathologic, immunohistochemical, and ultrastructural study of 15 cases.	Am J Surg Pathol 2005; 29:569-578	15	range 3-83	male to female ratio 2:1	chest pain, shortness of breath, neck or back pain, 4 patients had also constitutional symptoms such as fever, weight loss and weakness	tumor located in posterior mediastinum (6) / anterior mediastinum (6) / anterior-middle mediastinum (3)	complete surgical excision (10), partial excision followed by radiation (2), only radiation (3)	follow-up was available for 5 patients, 4 had local recurrence (follow-up from 1-3 years) and one patient died of tumor 6 months after diagnosis with liver metastases	4 cases had biopsy-proven metastases to hilar lymph node, lung, liver and epidural space
Al-Rajhi et al. [12]	Primary pericardial synovial sarcoma: a case report and literature review.	J Surg Oncol. 1999 Mar; 70(3):194-198	1	19	m	shortness of breath	large heart in chest X-ray, echocardiogramm indicated a pericardial mass and effusion, MRI revealed a 7 × 6 × 7.5 cm enhancing mass arising from pericardium	surgery with partial pericardectomy and radiation	free of disease 12 months after operation	first known pericardial synovial sarcoma
Kaira et al. [13]	Primary mediastinal synovial sarcoma: a report of 2 cases.	J Comput Assist Tomogr. 2008 Mar-Apr; 32(2):238-241.	2	64 / 58	f / m	right back pain and dysphagia / right back pain	heterogenous enhancing mass in the left posterior mediastinal space / posterior mediastinal mass	radiation and chemotherapy (ifosfamide and adriamycin) / radiation and chemotherapy (ifosfamide and adriamycin; gemcitabine and docetaxel; carboplatin and paclitaxel)	died 24 and 19 months after the initial diagnosis, respectively	in both cases no surgery, the neoplasm was unresectable

Immunohistochemically the tumor cells were positive for CAM 5.2, cytokeratin 7 (CK), bcl-2 and vimentin and negative for S100, CD34, CD99, desmin and SMA. With this findings (especially positive keratin and bcl-2) it was possible to exclude two entities that can most closely resemble monophasic synovial sarcoma in this location: Solitary fibrous tumor (CD34 positive) and malignant peripheral nerve sheath tumor (focally S-100 positive) [11].

CT scan of the tumor may demonstrate a heterogenous soft-tissue mass that occasionally contains calcium with attenuation slightly higher than that of muscle [14]. Invasion and infiltration of the surrounding tissue can be present on CT scan in patients with advanced stages of such tumors. In our patient the tumor size was > 5 cm but it did not compress or infiltrate any neighbouring organs. STSs typically grow in centrifugal fashion and can also compress surrounding structures [2].

Surgical resection with adequate safety margins represents the therapy of choice potentially being curative for STSs. Because of the atypical location and advanced size of > 5.0 cm it was decided to treat our patient also with adjuvant Ifosfamid/Adriamycin (doxorubicin) chemotherapy [13]. This is also recommended by Ferrari et al. who demonstrated effectiveness of adjuvant chemotherapy in such patients [15]. We also decided for a radiotherapy acknowledging the publication of Ferrari et al. which showed a risk reduction of a local recurrence from 50% to 7% when using radiation. It is also recommend in case of positive resection margins [12]. With this management, our patient is free of recurrence 16 months postoperatively.

## Abbreviations

CT: computed tomography
STS: soft tissue sarcomas
